# Directed Evolution of a Nonheme Diiron N-oxygenase AzoC for Improving Its Catalytic Efficiency toward Nitrogen Heterocycle Substrates

**DOI:** 10.3390/molecules27030868

**Published:** 2022-01-27

**Authors:** Ye Xu, Xiao-Fang Liu, Xin-Ai Chen, Yong-Quan Li

**Affiliations:** 1 Institute of Pharmaceutical Biotechnology, School of Medicine, Zhejiang University, Hangzhou 310058, China; xuye97@163.com (Y.X.); liuxiaofang@zju.edu.cn (X.-F.L.); 2 Zhejiang Provincial Key Laboratory for Microbial Biochemistry and Metabolic Engineering, Hangzhou 310058, China

**Keywords:** AzoC, 5-aminopyrimidine compounds, directed evolution, improved catalytic activity

## Abstract

The azoxy compounds with an intriguing chemical bond [-N=N^+^(-O^−^)-] are known to have broad applications in many industries. Our previous work revealed that a nonheme diiron N-oxygenase AzoC catalyzed the oxidization of amino-group to its nitroso analogue in the formation of azoxy bond in azoxymycins biosynthesis. However, except for the reported pyridine alkaloid azoxy compounds, most azoxy bonds of nitrogen heterocycles have not been biosynthesized so far, and the substrate scope of AzoC is limited to *p*-aminobenzene-type compounds. Therefore, it is very meaningful to use AzoC to realize the biosynthesis of azoxy nitrogen heterocycles compounds. In this work, we further studied the catalytic potential of AzoC toward nitrogen heterocycle substrates including 5-aminopyrimidine and 5-aminopyridine compounds to form new azoxy compounds through directed evolution. We constructed a double mutant L101I/Q104R via molecular engineering with improved catalytic efficiency toward 2-methoxypyrimidin-5-amine. These mutations also proved to be beneficial for N-oxygenation of methyl 5-aminopyrimidine-2-carboxylate. The structural analysis showed that relatively shorter distance between the substrate and the diiron center and amino acid residues of the active center may be responsible for the improvement of catalytic efficiency in L101I/Q104R. Our results provide a molecular basis for broadening the AzoC catalytic activity and its application in the biosynthesis of azoxy six-membered nitrogen catenation compounds.

## 1. Introduction

Natural azoxy compounds share a common functional group with the general structure [-N=N^+^(-O^−^)-], which have been found in bacteria, fungi, plants, and marine sponge [1,2]. According to the metabolism pathway, natural azoxy compounds are mainly involved in three classes including polyketides, shikimate-derived and amino acid-derived compounds. The special azoxy bond endows these compounds with multiple functionalities, especially with cytotoxic, nematocidal, and antimicrobial activity [3]. In the current industry, azoxy compounds are used widely as dyes, reducing agents, chemical intermediates, polymerization inhibitors, therapeutic agents, and energy materials [4,5,6,7]. Furthermore, the azoxy bond is proven to be an effective structural unit to improve the detonation performance of nitrogen catenation compounds [8]. However, at present, azoxy compounds, especially azoxy nitrogen catenation compounds, are mainly chemically synthesized, which is performed under harsh reaction conditions, such as high pressure, application of a metal catalyst and high temperature [6,9]. The biosynthesis of these azoxy compounds may lead to an option for reactions performed under mild conditions. To date, although pyridine alkaloid azoxy compounds from the natural origin have been reported [10,11,12], the biosynthesis of most azoxy nitrogen heterocycle compounds is still unknown. In addition, even if poly-nitrogen heterocycle compounds with azoxy bonds have not been discovered in nature, they have still attracted much attention due to their energy intensity [13].

**Figure 1:**
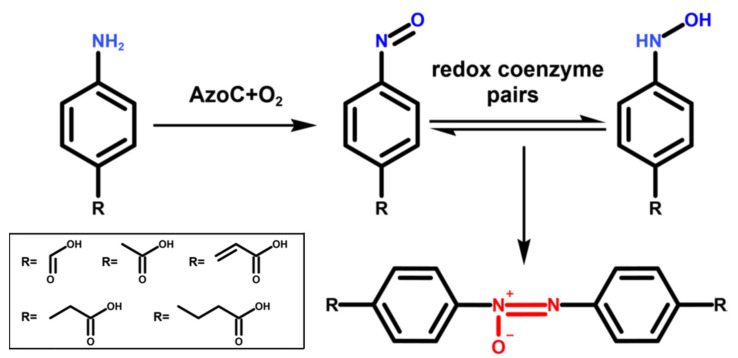
The general reaction catalyzed by AzoC and the substrate scope reported of AzoC [16].

Currently, the biosynthesis of azoxy compounds has been elucidated in only two biosynthetic pathways. One is the azoxy antibiotic valanimycin and the other is the yellow pigment azoxymycins [14,15]. The molecular mechanism of azoxy bond formation in azoxymycin’s biosynthesis was elucidated by our group through in vivo and in vitro reaction analysis [16]. For azoxymycins, the unique azoxy bond biosynthesis is controlled by a nonheme diiron N-oxygenase AzoC (an N-oxygenase; accession number: AKQ24642). First, the diiron center of the enzyme activates dioxygen to form oxygenated intermediate AzoC-P, which engages in the oxidization of amine to its nitroso analogue, and then redox coenzyme pairs facilitate the transformation of nitroso group into hydroxylamine conversely via the radical transient intermediates, which efficiently dimerize to azoxy bond (Figure 1). This catalytic process is composed of an enzymatic nitroso moiety-producing reaction and a non-enzymatic N–N bond coupling reaction. Our previous study showed AzoC exhibited the catalytic activity toward *p*-aminobenzene-type substrates (Figure 1) [16]. Whether AzoC can catalyze other types of substrates such as pyridine-type or pyrimidine-type is unknown. Further study of AzoC catalytic properties would broaden its potential use to synthesize more novel azoxy compounds.

Nowadays, biocatalysis is used as a practical and environmentally friendly alternative to traditional metallo- and organo- catalysis in chemical synthesis, both in the laboratory and on an industrial scale [17]. Using enzymes for transformations is well recognized due to their high reactivity at ambient conditions. Despite the clear advantages of enzymes, further improvement of their substrate scope and activity is paramount to their broader application on an industrial scale. Over the last several decades, directed evolution has become a method of choice in addressing these challenges [18]. The general procedure of directed evolution includes two main steps: (1) gene diversification by mutagenesis and (2) screening/selection to obtain variants with improved properties [19]. In order to reduce the size of mutation libraries and the workload of screening, the mutation library is usually constructed by saturation mutations at several specific sites. These sites are usually key amino acids that affect the catalytic activity or are located in the substrate binding pocket, and they are predicted by computer algorithms with the help of protein sequence, structure and function information [20,21,22,23,24]. Site-directed saturation mutagenesis is the most popular focused mutagenesis method in which a single amino acid residue can be substituted with all other amino acid residues. Saturation mutagenesis can help to produce protein forms that rarely appear in the lab or to study all the possible amino acids for a certain residue of the protein [25].

In this study, we first examined whether the catalytic potential of AzoC toward 5-aminopyrimidine and 5-aminopyridine-type substrates to form azoxy bonds. Then, we constructed a small mutant library by site-directed saturation mutagenesis based on analysis of active site residues and the sequence alignment to improve catalytic activity toward 2-methoxypyrimidin-5-amine. Furthermore, we investigated the effect of combined beneficial mutations together on the catalytic efficiency. The docking models of wild type AzoC and the mutant L101I/Q104R were constructed, respectively. The comparison of structural analysis suggested the reason for the improved catalytic activity. As the core structure of 5-aminopyrimidine compounds is similar to six-membered nitrogen catenation, this study provides a molecular basis for the biosynthesis of azoxy six-membered nitrogen catenation compounds.

## 2. Results

### 2.1. The Substrate Scope Extension of AzoC

**Figure 2:**
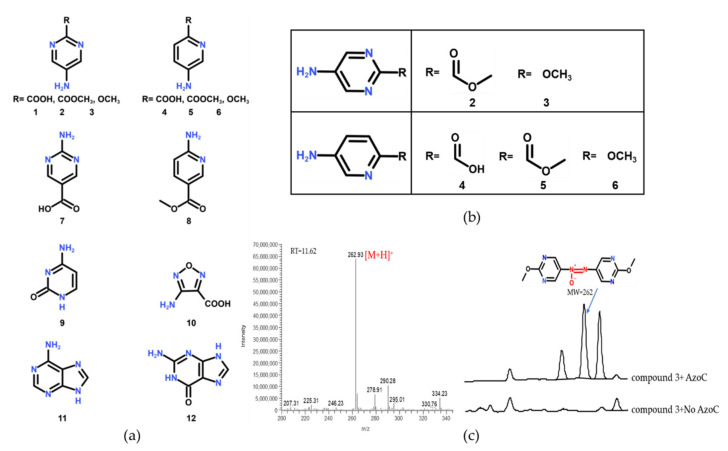
(**a**) Compounds tested with AzoC in this study. (**b**) The substrate scope extension of AzoC. (**c**) LC-MS analysis of AzoC in vitro reaction with 2-methoxypyrimidin-5-amine (compound 3) as the substrate. The molecular weight of the generated azoxy product is 262, which is in line with the theoretical value. The reaction was performed at 30 °C for overnight.

While the relevant enzymology of biogenic azoxy bond formation from N-heterocyclics remains currently uncharacterised [10,11,12], the substrate range of AzoC from *Streptomyces chattanoogensis* was reported to include a range of *p*-aminobenzene-type compounds [16]. Here, we first tested the substrate specificity of AzoC with a variety of compounds (Figure 2a), and the reaction mixture was analyzed by LC-MS. When the benzene ring was changed with the pyrimidine and pyridine rings, AzoC showed different catalytic activity according to different amino group positions. When the amino group was at the 2′ position of pyrimidine and pyridine rings (compound 7 and 8), AzoC showed no catalytic activity toward these compounds. Interestingly, AzoC exhibited catalytic activity toward compound 2–3 and compound 4–6 (Figure 2b,c and Figures S1,2 from Supplementary Materials), whose amino groups were at the 5′ position of pyrimidine and pyridine rings. The reason may be that the electron-withdrawing effect of the nitrogen atoms to amino groups was smaller when amino groups were at the 5′ position. Although the amino group of compound 1 was also at the 5′ position, AzoC showed almost no activity mainly because of the superimposed electron-withdrawing effect of two nitrogen atoms and the carboxyl group. The electron-withdrawing effect of two nitrogen atoms in the pyrimidine ring was stronger and the carboxyl group was also a stronger electron-withdrawing group compared with the ester group and methoxy group. When compounds 9–12 were used as the substrates, AzoC showed no activity, probably due to the huge difference in the skeleton structure of *p*-aminobenzene-type compounds. In this work, we found two kinds of new substrates catalyzed by AzoC, which were 5-aminopyrimidine and 5-aminopyridine–type compounds (Figure 2b,c and Figures S1,2). It was worth mentioning that their core structures were similar to that of the highly energetic substance 1,2,4,5-tetrazine-3,6-diamine [13]. Therefore, we used 5-aminopyrimidine compounds as substrates to perform directed evolution on AzoC to improve its catalytic activity toward 2-methoxypyrimidin-5-amine.

### 2.2. Mutational Analysis and Mutant Strain Construction

**Figure 3:**
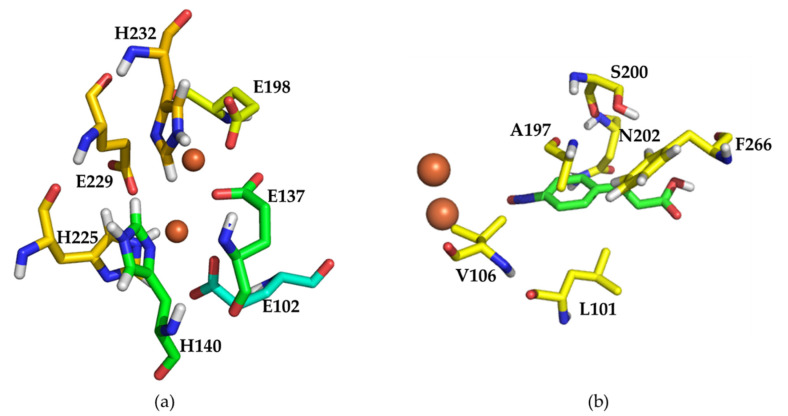
(**a**) The active site of wild type AzoC. The iron ions were shown as brown spheres. The two iron ions were coordinated with protein residues E102, E137, H140, E198, H225, E229, and H232. (**b**) Residues within 4Å of the substrate and around the benzene ring of 3-(4-nitrosophenyl) prop-2-enoic acid. The iron ions were shown as brown spheres. These two models were reported in the previous work [16]. The protein structure of wild type AzoC was constructed with Modeller 9.18 using AurF (PDB ID: 3chh.1A) as template.

In order to select appropriate mutation sites to construct a mutation library, we conducted mutation analysis on active amino acid sites based on previous results [16]. The two iron ions were coordinated with residues E102, E137, H140, E198, H225, E229, and H232 to form the active site of wild type AzoC (Figure 3a). Then, alanine scanning was performed on these seven residues in the active site, resulting in the loss of AzoC activity, respectively (Table S1 from Supplementary Materials). The results proved that these seven residues were responsible for the catalytic activity of AzoC. To further test the conservation of these seven amino acids as well as D136 and D228, we mutated them into amino acids with the same charge and similar structure (Table S1). Similarly, these mutations also caused enzyme inactivation. It showed that these key amino acids were very critical to the catalytic activity of AzoC.

Based on the docking model reported in the previous work [16], we found residues of L101, V106, A197. S200, N202, and F266 directly surround the benzene ring of the substrate due to the replacement of benzene ring with a pyrimidine (Figure 3b). Alanine mutation of these residues also inactivated the AzoC activity (Table S2). Although these residues were not directly engaged in the active center, the mutation of these residues to alanine may affect the substrate binding and the structure of the active center because of its proximity to the substrate and diiron center. Consequently, these six residues were used for construction of site-directed saturation mutagenesis. At the same time, we constructed three mutants, Q104R, Q111E and I199T through sequence alignment of AzoC, AurF (an N-oxygenase; accession number: CAE02601), and CmlI (an aryl amine oxidase; accession number: 5HYH_A). We found these three sites were located in the iron binding domain of these three proteins, and they were charged amino acids or polar amino acid residues in AurF and CmlI (Figure S3). Besides, the 5-aminopyrimidine substrate (2-methoxypyrimidin-5-amine) had more polarity than an equivalent aromatic ring.

### 2.3. Beneficial Mutations with Improved Catalytic Efficiency toward 5-Aminopyrimidine-Type Compounds

**Figure 4:**
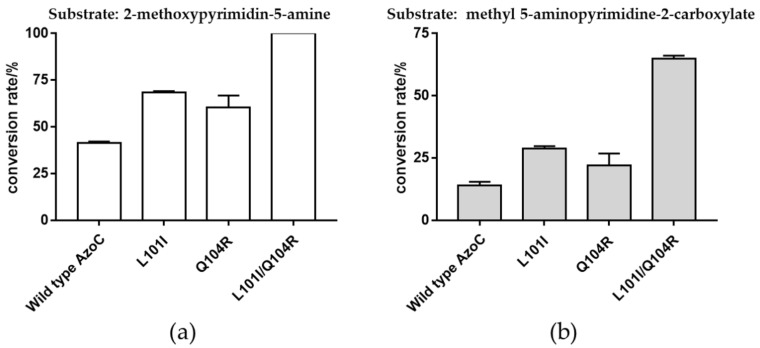
(**a**) Comparison of 2-methoxypyrimidin-5-amine conversion rate between wild type AzoC and the mutants L101I, Q104R and L101I/Q104R. (**b**) Comparison of methyl 5-aminopyrimidine-2-carboxylate conversion rate between wild type AzoC and the mutants L101I, Q104R and L101I/Q104R. All reactions were performed with purified protein at 30 °C for overnight in two parallel, respectively. SD bars were shown in the figure.

The effect of mutations on activity of wild type AzoC was first determined through detection of newly generated azoxy compounds in crude cell lysate by HPLC (Tables S2 and S3). Then we therefore performed a rough comparison of the activity of the mutants based on the peak area of the azoxy product using crude cell lysate reactions. We found that four mutants may have improved activity, which were L101I, L101V, A197S, and Q104R (Tables S2 and S3). Then the candidate four mutants were selected to further validate and accurately compare their activity with the wild type AzoC through in vitro reactions using purified protein. Combining these two results of the activity comparison (Tables S2, S3 and Figure S4, Figure 4a), it showed that two variants L101I and Q104R indeed had improved catalytic activity toward 2-methoxypyrimidin-5-amine due to the improved conversion rate of the substrate (Figure 4a). The apparent synergistic effect of these two beneficial mutations was further investigated through construction of a double mutant L101I/Q104R. As we expected, the combined mutant L101I/Q104R showed a superposition effect on improving catalytic activity for the azoxy bond formation toward the substrate of 2-methoxypyrimidin-5-amine (Figure 4a). Similarly, when challenged with the substrate 5-aminopyrimidine-2-carboxylate, both L101I and Q104R exhibited relatively small increased rates compared to wild type AzoC, while that of L101IQ104R was increased significantly (Figure 4b). As with 2-methoxypyrimidine-5-amine, these results indicated that both single mutants, but especially the double mutant, exhibited catalytic activities that enhanced azoxy bond formation from 5-amino-pyrimidine substrates.

We also determined kinetic parameters of the wild type AzoC and the mutants L101I, Q104R, and L101I/Q104R, respectively, with 2-methoxypyrimidine-5-amine as the test substrate. As shown in Table 1, the K_m_ values of all beneficial mutants were lower than that of the wild type AzoC, suggesting that these mutants had better affinity with the substrate. The k_cat_ of L101I and L101I/Q104R was higher than that of the wild type AzoC, demonstrating increased turnover numbers. Further, the catalytic efficiency (k_cat_ /K_m_) of L101I, Q104R and L101I/Q104R were increased by 2.19-, 1.38-, and 3.02-fold, respectively, as compared to that of wild type AzoC (Table 1). These results indicated that L101I and L101I/Q104R had higher catalytic activity probably due to the increased turnover numbers and better affinity with the substrate, while the improvement of substrate affinity was the possible reason for increased catalytic activity of Q104R.

### 2.4. Structural Analysis of Wild Type AzoC and the Double Mutant L101I/Q104R

**Figure 5:**
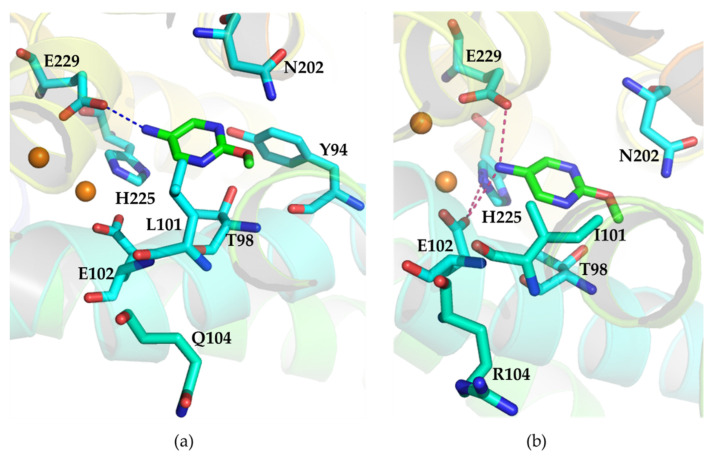
(**a**) Docking of 2-methoxypyrimidin-5-amine into wild type AzoC. The iron ions were shown as brown spheres. The hydrogen bond was shown as blue dashed line. The polar interaction with residues N202, Y94, T98 was not shown in the figure. (**b**) Docking of 2-methoxypyrimidin-5-amine into the mutant L101I/Q104R. The iron ions were shown as brown spheres. The hydrogen bonds were indicated as warm pink dashed lines. The polar interaction with residues N202, T98 was not shown in the figure.

To better understand the structural basis of the increased catalytic activity of double mutant L101I/Q104R, we docked 2-methoxypyrimidin-5-amine into wild type AzoC and the mutant L101I/Q104R using AurF (PDB ID:3chh.1A) as template. In AzoC, the two iron ions were coordinated with protein residues E102, E137, H140, E198, H225, E229, and H232 to form its active site [16]. This found that E102 formed an additional hydrogen bond with H225 in the L101I/Q104R mutant docking model, but not in wild type AzoC docking model (Figure 5). This outcome suggested that the interaction between the key residues in the active center was stronger in L101I/Q104R, which may make the entire active center more stable. In addition, the substrate formed one hydrogen bond only with AzoC E229, while two hydrogen bonds between the substrate with E229 and E102 of L101I/Q104R, respectively (Figure 5). This indicated that the interaction of 2-methoxypyrimidin-5-amine with the active center was stronger. Furthermore, the distance between the nitrogen atom on the amino group of the substrate and Fe(I), Fe(II) respectively were 4.3Å and 3.1Å in the L101I/Q104R docking model, which were 5.7Å and 4.5Å in the wild type AzoC docking model (Figure S5). The shorter distance of the substrate to iron ions may be beneficial to the oxidation of the amino group by the diiron center.

## 3. Discussion

Compounds containing azoxy bonds have many bioactivities such as cytotoxic, nematicidal, and antimicrobial activity [3], which have attracted much attention. Improving and expanding the functions of N-oxygenase AzoC is key to forming the new azoxy compounds. A common feature of the reactions catalyzed by these nonheme diiron enzymes is that the diiron center of the enzyme activates dioxygen to form reactive O_2_ adducts such as diiron(II,III)-superoxo, diiron(III)-peroxo, diiron(III,IV)-oxo, and diiron(IV)-oxo species, which carry out the substrate oxidation [26]. Therefore, the shorter distance of the substrate to iron ions may be beneficial to the oxidation of the amino group. In AzoC, the two iron ions were coordinated with protein residues E102, E137, H140, E198, H225, E229, and H232 to form its active site [16]. Although the mutation of L101I and Q104R do not directly form new interactions with the substrate, they may have changed the shape of the active site and substrate binding pocket due to an additional hydrogen bond formation between E102 and H225 in L101I/Q104R (Figure 5). When L101 is mutated to an isoleucine, the substrate may enter the active site more easily, because the side chain of isoleucine reduced the hindrance to the substrate (Figure 5). Unlike L101I, Q104R these close mutations, which may affect the substrate binding pocket and the active site, distal mutations also have an impact on enzyme catalysis by altering the enzyme conformational dynamics [27,28,29]. Common methods to make distal mutations are typically random mutagenesis, such as error-prone PCR and DNA shuffling. Consequently, we propose to focus on the distal residues to acquire mutants with further increased catalytic activity in the future work.

When the amino group was at the 2′ position of pyrimidine and pyridine rings (compound 7 and 8), both beneficial mutants and wild type AzoC showed no activity toward these compounds due to the strong electron-withdrawing effect of the nitrogen atoms in these nitrogen-containing rings. However, the electron-withdrawing effect of six-membered nitrogen catenation (1,2,4,5-tetrazine-3,6-diamine) is stronger than 2-aminopyrimidine compounds due to four nitrogen atoms in the ring, so it is more difficult for the enzyme to catalyze these substrates to form an azoxy bond. Therefore, strategies aimed at further increasing the substrate range and oxidizability potential of the AzoC enzyme should be explored. It seems feasible to add some functional molecules to help enzyme catalysis. In 2018, it was reported that the introduction of an exogenous small molecule into a catalytic system of an artificial P450 peroxygenase activated normally H_2_O_2_-inert P450s [30]. The other method can be de novo protein design. Man-made proteins can reproduce selected functions and catalytic activities of natural metalloenzymes [31,32,33]. One example was reported in 2012, which is that the created protein to catalyze the O_2_-dependent, two-electron oxidation of hydroquinones, has been reprogrammed to catalyze the selective N-hydroxylation of arylamines by remodeling the substrate access cavity and introducing a critical third His ligand to the metal-binding cavity [32].

## 4. Materials and Methods

Reagents were purchased from Sigma-Aldrich (Shanghai, China), OKA (Beijing, China), Bidepharm (Shanghai, China) and Aladdin Bio-Chem Technology (Shanghai, China). The commercial, chemically competent *Escherichia coli* DH5α was used for routine DNA manipulation (Tsingke Biotech, Hangzhou, China), and *Escherichia coli* BL21(DE3) was used as host for protein expression (Weidi Biotechnology Co., Shanghai, China). The plasmid pET28a-*azoC* was constructed by our lab.

### 4.1. Protein Expression and Purification

*Escherichia coli* BL21(DE3) was transformed by the plasmid harboring each gene and grown in 200 mL of lysogeny broth (LB) at 37 °C with shaking at 220 rpm until an OD_600_ of 0.5–0.6 was reached. The culture was added with 0.1–0.2 mM isopropyl β-D-1-thiogalactopyranoside and 1 mM ferric chloride for induction 16–20 h at 16 °C with shaking at 160 rpm. Cells were collected and disrupted in buffer I (20 mM Tris, pH 8.0, 250 mM NaCl, and 10 mM imidazole) by sonication. The suspension was centrifuged (10,000 rpm, 20 min, 4 °C) twice, and the resulting supernatant was purified on Ni^2+^-nitrilotriacetic acid resin and eluted with buffer II (20 mM Tris, pH 8.0, 250 mM NaCl, and 250 mM imidazole). Then, the protein was concentrated and desalted on a 10-kDa YM-3 column with 20 mM Tris buffer (pH 7.5). Protein concentration was determined with the Bradford assay.

### 4.2. In Vitro Reaction System and LC-MS Analysis

In vitro biochemical reactions were performed by incubating the 5–10 μM purified enzyme with 100–250 μM substrates, 0.1 mM NADH, and 0.75 mM PMS in 20mM HEPES buffer (pH 7.5) at 30 °C for overnight. The reactions were stopped by the addition of an equal volume of methanol. The reaction mixture was analyzed by HPLC. Analysis of in vitro reactions was carried out on Agilent 1260 infinity system (Agilent, Beijing, China) with a DAD detector at 320 nm. The column used was Agilent Extend-C18 (5 μm, 4.6×150 mm). Mobile phase A was 0.1% formic acid in water, and mobile phase B was acetonitrile. Flow rate was 1 mL/min^−1^. During the analysis procedure, mobile phase B was increased from 5 to 75% in 20 min.

### 4.3. Mutation Library Construction

AzoC mutant libraries were created by site-directed mutagenesis and site-directed saturation mutagenesis libraries through plasmid PCR using primers containing the mutation of interest (Tables S4 and S5) and plasmid pET28a-*azoC* as the template. The purified PCR products were digested by *Dpn*I and then transformed into *E. coli* DH5α for DNA sequencing. The confirmed mutant plasmids were transformed into *E. coli* BL21 (DE3) for expression and activity test.

### 4.4. The Catalytic Activity Assay of Crude Cell Lysates and the Purified Protein

Single colonies of *E. coli* BL21 (DE3) harboring plasmids were picked from LB agar plate into 1 mL of self-induced medium (LB supplemented with 10 g/L lactose, 0.5 g/L glucose, 1 mM ferric chloride and 100 ug/mL kanamycin) and grown at 37 °C and 220 rpm for 5–6 h. Then, bacterial solution was cultured overnight at 25 °C. After centrifugation of the cultures at 15,000 rpm for 2–3 min, the resulting cell pellets were resuspended in 0.1 mL of cell lysis reaction solution (50 mM Tris, 150 mM NaCl, 1.5% Tween-20, 1.5 g/L lysozyme, 1.5 mM PMSF, 0.5 mM PMS and 0.1 mg/mL 2-methoxypyrimidin-5-amine, pH 7.4), and incubated at 30 °C, 250 rpm for 8 h. The reactions were stopped by the addition of 0.1 mL of methanol. The reaction mixtures were analyzed by HPLC. The protein of active mutants was further purified to determine their activity toward 2-methoxypyrimidin-5-amine. The catalytic activity of the interest protein was calculated through substrate conversion rate of in vitro reactions.

### 4.5. Kinetic Data Analysis of Wild Type AzoC and the Mutant L101I, Q104R, L101I/Q104R

For kinetic analysis, the concentration of wild type AzoC and the mutants L101I, Q104R, L101I/Q104R were kept constantly at 5.2 μM (AzoC), 3 μM (the mutants), respectively, and the concentration of substrate 2-methoxypyrimidin-5-amine was varied from 0.05 to 2 mM. The reactions were incubated at 30 °C for 1 h and stopped by the addition of equal volume of methanol. The reaction mixtures were analyzed by HPLC, and the obtained initial velocity data were fitted to the Michaelis–Menten equation by using GraphPad Prism version 7.0 (GraphPad, San Diego, CA, USA).

### 4.6. Molecular Modeling and Docking Analysis of Wild Type AzoC and the Mutant L101I/Q104R

The protein structures of AzoC and L101I/Q104R were set up with the molecular modeling programs SWISS-MODEL (https://swissmodel.expasy.org/, accessed on 24 October 2021). The crystallographic structure of AurF (PDB ID:3chh.1A) was used as tertiary structural template, which showing 37% sequence identity to AzoC. The generated models (AzoC and L101I/Q104R) were used for docking of 2-methoxypyrimidin-5-amine, respectively. Graphical User Interface program AutoDock Tools version 1.5.6 (The Scripps Research Institute, La Jolla, CA, USA) was used to prepare the ligand and receptor. AutoGrid version 1.5.6 (The Scripps Research Institute) was used for the preparation of the grid map using a grid box. For AzoC, grid box parameters (x, y, z): (−6.423, 0.145, −4.139); size (15, 15, 15). For L101I/Q104R, grid box parameters (x, y, z): (−6.419, 0.131, −4.155); size (15, 15, 15). AutoDock/Vina version 1.1.2 (The Scripps Research Institute) was employed for docking using protein and ligand information along with grid box properties in the configuration file. During the docking procedure, both the protein and ligands are considered rigid. The results of docking were analyzed by PYMOL version 1.7.0.0 (Schrödinger, New York, NY, USA).

## 5. Conclusions

In conclusion, this work first broadened the confirmed substrate scope of wild type AzoC to include 5-aminopyrimidine and 5-aminopyridine–type compounds. Secondly, directed evolution was performed on AzoC to improve the catalytic efficiency toward 5-aminopyrimidine compounds. After mutational analysis, six residues L101, V106, A197, S200, N202 and F266 were selected for site-directed saturation mutagenesis. We also constructed Q104R, Q111E, I199T mutants by the sequence alignment of AzoC, AurF, and CmlI. Among them, variants L101I and Q104R exhibited the improved catalytic activity toward 2-methoxypyrimidin-5-amine. The double mutant L101I/Q104R had the best catalytic activity toward 2-methoxypyrimidin-5-amine, which indicated that the combination of these two beneficial mutation sites had a positive synergistic effect on enzyme activity. The k_cat_/K_m_ values of the mutant L101I, Q104R, L101I/Q104R were improved by 2.19, 1.38 and 3.02 folds, respectively, compared to AzoC. Similar outcomes were recorded with a second tested 5-aminopyrimidine substrate. The structural analysis showed that the shorter distance of the substrate to Fe(I), Fe(II) and the residues of active center may be responsible for higher catalytic efficiency toward 2-methoxypyrimidin-5-amine. This work provided a molecular basis for the biosynthesis of azoxy six-membered nitrogen catenation compounds.

## Figures and Tables

**Table 1:** The kinetic parameters of AzoC and the mutants L101I, Q104R, L101I/Q104R.

Enzyme	Substrate: 2-methoxypyrimidin-5-amine			
	K_m_ (mM)	k_cat_ (s^−1^)	k_cat_/K_m_ (M^−1^s^−1^)	Improvement
Wild type	0.65 ± 0.12	0.018 ± 0.0014	27.7	1
L101I	0.51 ± 0.059	0.031 ± 0.0013	60.8	2.19-fold
Q104R	0.42 ± 0.082	0.016 ± 0.0012	38.1	1.38-fold
L101I/Q104R	0.55 ± 0.060	0.046 ± 0.0019	83.6	3.02-fold

## Data Availability

Not available.

## Supplementary Materials

The Supplementary Materials are available online. Figures S1 and S2: LC-MS analysis of AzoC enzyme reaction with compound 2-6 as substrates; Figure S3: Sequence alignment of AzoC, AurF and CmlI; Figure S4: Comparison of the enzyme activity toward 2-methoxypyrimidine-5-amine between wild type AzoC and the mutants L101V, A197S; Figure S5: Docking of 2-methoxypyrimidin-5-amine into wild type AzoC and L101I/Q104R; Tables S1, S2 and S3: The activity of the crude cell lysates of the mutants; Tables S4 and S5: The primers used to construct mutants.
