# Supplementary material for: Directed Evolution of a Nonheme Diiron N-oxygenase AzoC for Improving Its Catalytic Efficiency toward Nitrogen Heterocycle Substrates
Source: Molecules. 2022 Jan 27;27(3):868. doi: 10.3390/molecules27030868 (PMC8840469; doi:10.3390/molecules27030868)
Supplement: Supplementary file 1 [file molecules-27-00868-s001.zip › molecules-1556231-supplementary.pdf]

molecules

Supplementary Materials

Directed evolution of a nonheme diiron N-oxygenase AzoC for improving its catalytic efficiency toward nitrogen heterocycle substrates

Ye Xu[1,2], Xiao-Fang Liu[1,2], Xin-Ai Chen[1,2]* and Yong-Quan Li [1,2]*

[1] Institute of Pharmaceutical Biotechnology, School of Medicine, Zhejiang University, Hangzhou 310058, China

[2] Zhejiang Provincial Key Laboratory for Microbial Biochemistry and Metabolic Engineering, Hangzhou 310058, China

* Correspondence: Y.Q.L.: lyq@zju.edu.cn; Tel. 86-571-88206632; X.A.C.: biolab@zju.edu.cn; Tel:86-571-88208569.

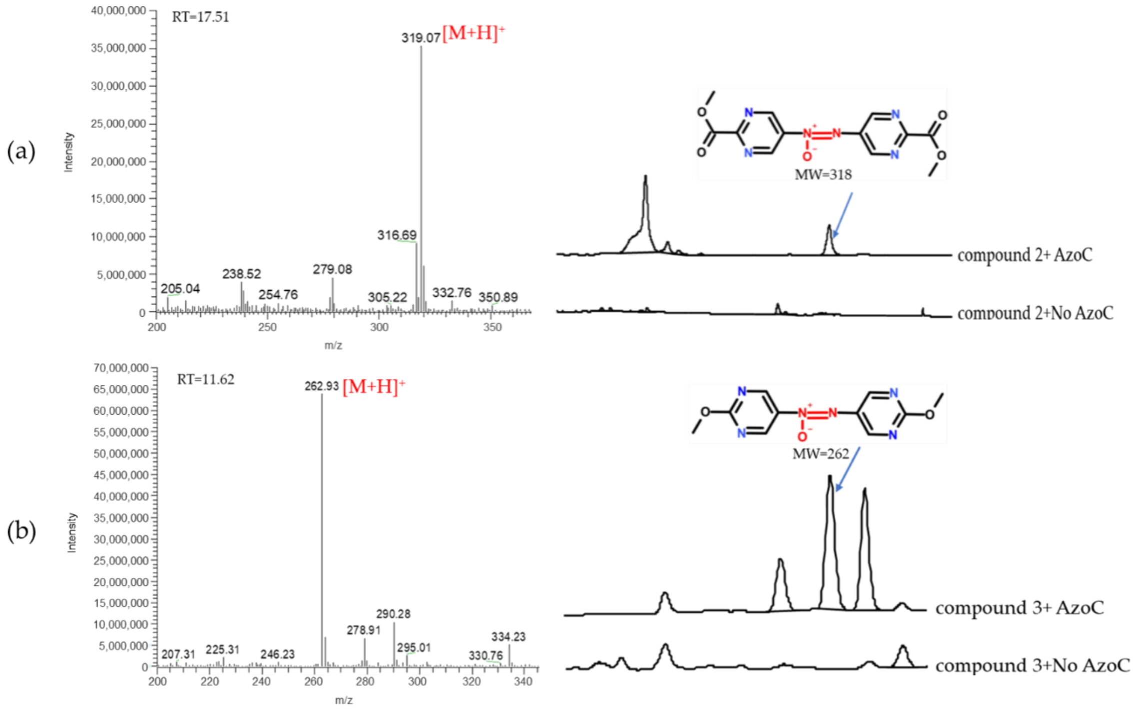

Figure S1. (**a**) LC-MS analysis of AzoC in vitro reaction with methyl 5-aminopyrimidine-2-carboxylate (compound 2) as the substrate. The molecular weight of the generated azoxy product is 318, which is in line with the theoretical value. (**b**) LC-MS analysis of AzoC in vitro reaction with 2-methoxypyrimidin-5-amine (compound 3) as the substrate. The molecular weight of the generated azoxy product is 262, which is in line with the theoretical value. All reactions were performed at 30 °C for overnight.

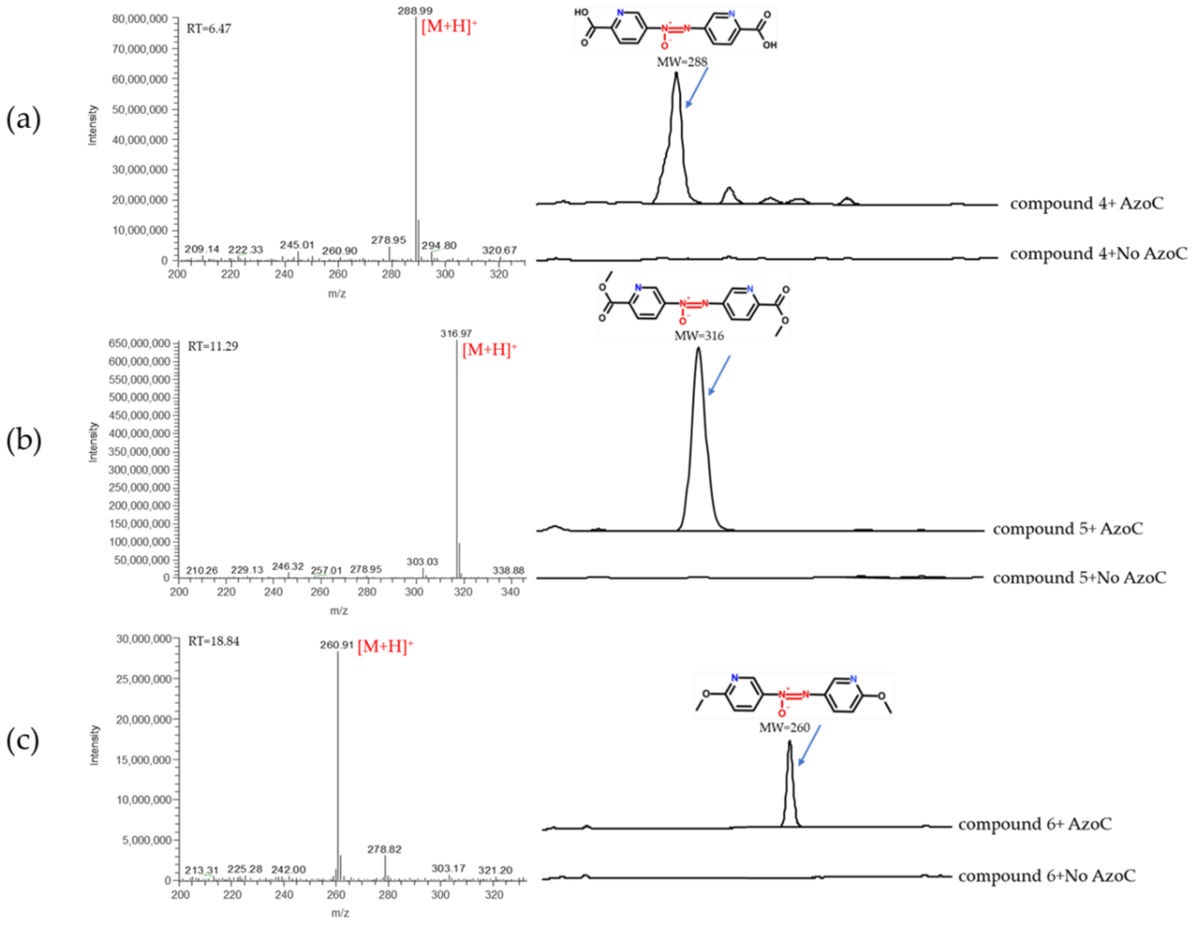

Figure S2. (**a**) LC-MS analysis of AzoC in vitro reaction with 5-aminopyridine-2-carboxylic acid (compound 4) as the substrate. The molecular weight of the generated azoxy product is 288, which is in line with the theoretical value. (**b**) LC-MS analysis of AzoC in vitro reaction with methyl 5-aminopyridine-2-carboxylate (compound 5) as the substrate. The molecular weight of the generated azoxy product is 316, which is in line with the theoretical value. (**c**) LC-MS analysis of AzoC in vitro reaction with 2-methoxy-5-aminopyridine (compound 6) as the substrate. The molecular weight of the generated azoxy product is 260, which is in line with the theoretical value. All reactions were performed at 30 °C for overnight.

```
AzoC  1    MSSRAPEELTGVQSPELPAYDPDDQAENAVIARLAGNWHRRAAV--KREEPNLADLFELARDDYPERILPFRDHPTFRAL  78
AurF  1    MREEQPHLATTWAA---RGWVEEEGIGSATLGRLVRAWPRRAAVVNKADILDEWADYDTLVPDYPLEIVPFAEHPLFLAA  77
CmlI  1    ---------------HHHHHHHHAIAENAVINRLVGNWHRRAAV--KREEPDVYALFDPGRPDFREDMIPFRGHPIWERL  63

AzoC  79   PPEDRARLLSWAWISYNRTTVLLEGQIVNPAFQLGLDGEFPQPVSELMQRSLAQAMVDEQYHTLMHLNASAVTRRRRGEA  158
AurF  78   EPHQRQRVLTGMWIGYNERVIATEQLIAEPAFDLVMHGVFPGSDDPLIRKSVQQAIVDESFHTYMHMLAIDRTRELRK--  155
CmlI  64   SDETRSRLLSWGWVAYNRNTVLIEQRIANPAFELVIGGAYPGLGGQQLELAVAQAMVDEQYHTLMHINGSAVTRRMRRSD  143

AzoC  159  FADAALPKPLVVREHEAR--LASCANERERRLTTLAFATVAEISINAYLNLIADDKEIQPVNSATVRIHNRDEYCHASIS  236
AurF  156  ISERP-PQPELVTYRRLRRVLADMPEQWERDIAVLVWGAVAETCINALLALLARDATIQPMHSLITTLHLRDETAHGSIV  234
CmlI  144  FSDRVLPDSHITTIHQEH--LDRCEEPWQRSLTTLGFATVAEISINAYLDLLADDQEIQVVNSTTVKLHNRDEYCHASIS  221

AzoC  237  AVLAEQVHHTLDDGERRYFLQSLVAGLEAFVGNDFMAWHRIMDEAGIRGGHEMLDDIQHAGGRKRLVQDFSGLRKLVERL  316
AurF  235  VEVVRELYARMNEQQRRALVRCLPIALEAFAEQDLSALLLELNAAGIRGAEEIVGDLRSTAGGTRLVRDFSGARKMVEQL  314
CmlI  222  GEMMKQVYEALPADRRRFLLEKVVAGLEAFVAPDFTTWESIVAFEGVPGWEKAAAEVREAQGGTHLVQDHSGIHTLLTEM  301

AzoC  317  DAVDDLDFDWSRSVTGSDAVSPTR  340
AurF  315  GLDDAVDFDFPERPDWSPHTP--R  336
CmlI  302  DVLDQVEFGWGTTVT--------R  317
```

Figure S3. Sequence alignment of AzoC (an N-oxygenase; accession number: AKQ24642), AurF (an N-oxygenase; accession number: CAE02601), and CmlI (an aryl amine oxidase; accession number: 5HYH_A). Iron binding motifs were marked in red. The AzoC mutants Q104R, Q111E, I199T were highlighted in blue, yellow and grey.

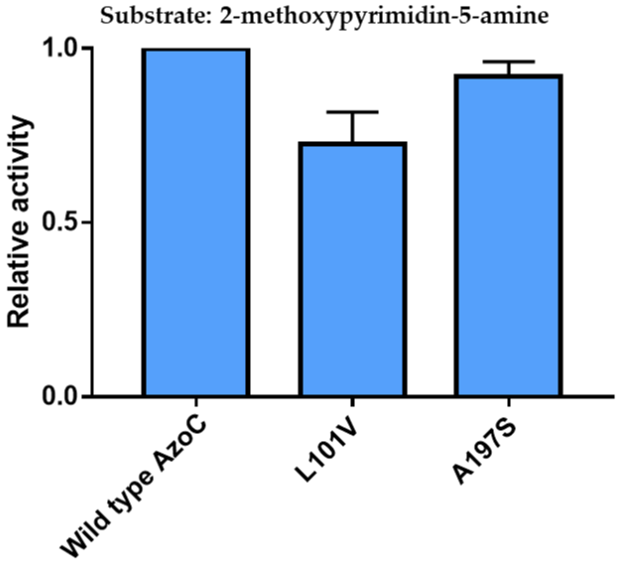

Figure S4. Comparison of the enzyme activity toward 2-methoxypyrimidine-5-amine between wild type AzoC and the mutants L101V, A197S. All reactions were performed with purified protein at 30 °C for overnight in two parallel, respectively. SD bars were shown in the figure.

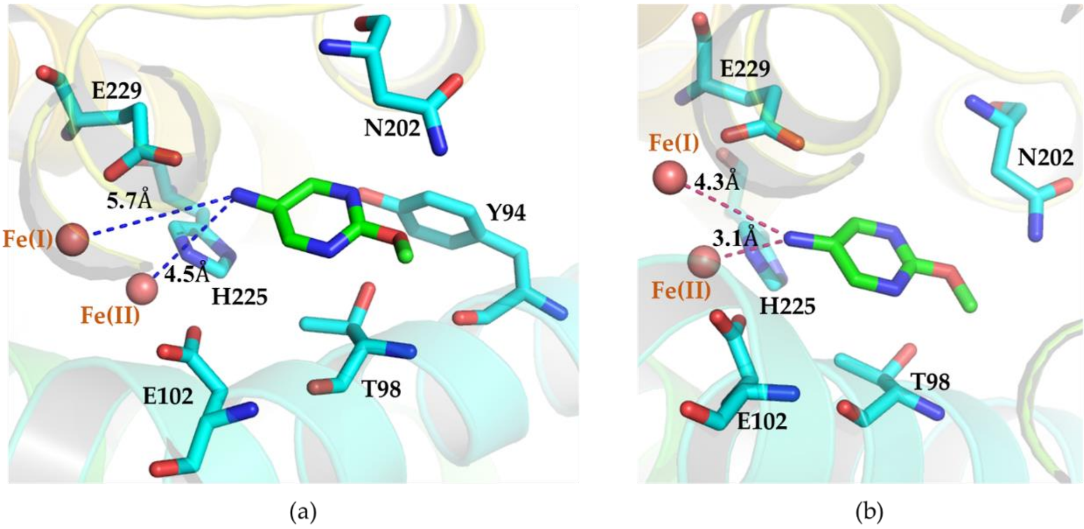

Figure S5. (**a**) Docking of 2-methoxypyrimidin-5-amine into wild type AzoC. The iron ions were shown as brown spheres. The polar interaction with residues N202, Y94, T98 was not shown in the figure. The distance between the nitrogen atom on the amino group of the substrate and Fe(I), Fe(II) respectively were 5.7Å and 4.5Å. (**b**) Docking of 2-methoxypyrimidin-5-amine into the mutant L101I/Q104R. The iron ions were shown as brown spheres. The polar interaction with residues N202, T98 was not shown in the figure. The distance between the nitrogen atom on the amino group of the substrate and Fe(I), Fe(II) respectively were 4.3Å and 3.1Å.

Table S1. The activity of the mutation in or near active site.

Mutation	Activity
E102A	-
E137A	-
E198A	-
E229A	-
H140A	-
H225A	-
H232A	-
H140R	-
H225R	-
H232R	-
E102D/E229D	-
E102D/E137D	-
E102D/E137D/E229D	-
E102D/E137D/E198D/E229D	-
D136E	-
D228E	-

Note: "**-**" means "No activity".

Table S2. The activity of the crude cell lysate of the mutation in L101, V106, A197, S200, N202 and F266 toward 2-methoxypyrimidin-5-amine.

Mutation	Activity
Negative control	-
Wild type AzoC	+
L101G	-
L101A	-
L101V	++
L101M	+
L101I	++
L101S	-
L101T	+
L101C	+
L101P	-
L101N	-
L101Q	-
L101K	-
L101R	-
L101H	-
L101D	-
L101E	-
L101F	+
L101Y	+
L101W	-
V106G	-
V106A	-
V106L	-
V106M	+
V106I	+
V106S	-
V106T	+
V106C	+
V106P	-
V106N	-

V106Q	-
V106K	-
V106R	-
V106H	-
V106D	-
V106E	-
V106F	-
V106Y	-
V106W	-
A197G	+
A197V	+
A197L	-
A197M	-
A197I	+
A197S	++
A197T	-
A197C	-
A197P	-
A197N	-
A197Q	-
A197K	-
A197R	-
A197H	-
A197D	-
A197E	-
A197F	-
A197Y	-
A197W	-
S200G	-
S200V	-
S200L	-
S200M	-
S200I	-
S200A	-
S200T	+

S200C	-
S200P	-
S200N	-
S200Q	-
S200K	-
S200R	-
S200H	-
S200D	-
S200E	-
S200F	-
S200Y	-
S200W	-
N202G	-
N202V	-
N202L	-
N202M	-
N202I	-
N202A	-
N202T	-
N202C	-
N202P	+
N202S	-
N202Q	+
N202K	-
N202R	-
N202H	-
N202D	-
N202E	-
N202F	-
N202Y	-
N202W	-
F266G	-
F266V	+
F266L	-
F266M	+

F266I	+
F266A	-
F266T	-
F266C	-
F266P	-
F266S	-
F266Q	-
F266K	-
F266R	-
F266H	-
F266D	-
F266E	-
F266N	-
F266Y	-
F266W	-

Note: "+" means that the mutant has activity toward the substrate, but the peak area of the azoxy product is similar to or smaller than that of wild type AzoC. "++" means that the mutant has activity toward the substrate, and the peak area of the azoxy product is bigger than that of wild type AzoC. "-" means that the mutant has no activity toward the substrate. "Negative control" is the crude cell lysate of *Escherichia coli* BL21(DE3) transformed by the plasmid harboring no target gene.

Table S3. The activity of the crude cell lysate of Q104R, Q111E and I199T toward 2-methoxypyrimidin-5-amine.

Mutation	Activity
Negative control	-
Wild type AzoC	+
Q104R	++
Q111E	-
I199T	+

Note: "+" means that the mutant has activity toward the substrate, but the peak area of the azoxy product is similar to or smaller than that of wild type AzoC. "++" means that the mutant has activity toward the substrate, and the peak area of the azoxy product is bigger than that of wild type AzoC. "-" means that the mutant has no activity toward the substrate. "Negative control" is the crude cell lysate of *Escherichia coli* BL21(DE3) transformed by the plasmid harboring no target gene.

Table S4. The primers for site-directed saturation mutation.

Name of primers	Sequence (5' to 3')
xy-101Ala-R	cgcaccacggtgctgGCGgaggggcagatcgtc
XY-101Ala-F	gacgatctgcccctcCGCcagcaccgtggtgcg
xy-101Gly-R	cgcaccacggtgctgGGGgaggggcagatcgtc
XY-101Gly-F	gacgatctgcccctcCCCcagcaccgtggtgcg
xy-101Val-R	cgcaccacggtgctgGTGgaggggcagatcgtc
XY-101Val-F	gacgatctgcccctcCACcagcaccgtggtgcg
xy-101Ile-R	cgcaccacggtgctgATCgaggggcagatcgtc
XY-101Ile-F	gacgatctgcccctcGATcagcaccgtggtgcg
xy-101Ser-R	cgcaccacggtgctgTCCgaggggcagatcgtc
XY-101Ser-F	gacgatctgcccctcGGAcagcaccgtggtgcg
xy-101Cys-R	cgcaccacggtgctgTGCgaggggcagatcgtc
XY-101Cys-F	gacgatctgcccctcGCAcagcaccgtggtgcg
xy-101Lys-R	cgcaccacggtgctgAAGgaggggcagatcgtc
XY-101Lys-F	gacgatctgcccctcCTTcagcaccgtggtgcg
xy-101His-R	cgcaccacggtgctgCACgaggggcagatcgtc
XY-101His-F	gacgatctgcccctcGTGcagcaccgtggtgcg
xy-101Glu-R	cgcaccacggtgctgGAGgaggggcagatcgtc
XY-101Glu-F	gacgatctgcccctcCTCcagcaccgtggtgcg
xy-101Phe-R	cgcaccacggtgctgCTTgaggggcagatcgtc
XY-101Phe-F	gacgatctgcccctcGAAcagcaccgtggtgcg
xy-101Tyr-R	cgcaccacggtgctgTACgaggggcagatcgtc
XY-101Tyr-F	gacgatctgcccctcGTAcagcaccgtggtgcg
xy-101Trp-R	cgcaccacggtgctgTGGgaggggcagatcgtc
XY-101Trp-F	gacgatctgcccctcCCAcagcaccgtggtgcg
xy-101-R	cgcaccacggtgctgNNKgaggggcagatcgtc
XY-101-F	gacgatctgcccctcMNNcagcaccgtggtgcg
xy-106ILE-R	cgaggggcagatcATCaatcctgcgttcc
XY-106ILE-F	ggaacgcaggattGATgatctgcccctcg
xy-106GLY-R	cgaggggcagatcGGTaatcctgcgttcc
XY-106GLY-F	ggaacgcaggattACCgatctgcccctcg
xy-106HIS-R	cgaggggcagatcCACaatcctgcgttcc
XY-106HIS-F	ggaacgcaggattGTGgatctgcccctcg
xy-106CYS-R	cgaggggcagatcTGCaatcctgcgttcc
XY-106CYS-F	ggaacgcaggattGCAgatctgcccctcg
xy-106ALA-R	cgaggggcagatcGCCaatcctgcgttcc
XY-106ALA-F	ggaacgcaggattGGCgatctgcccctcg
xy-106LEU-R	cgaggggcagatcCTGaatcctgcgttcc
XY-106LEU-F	ggaacgcaggattCAGgatctgcccctcg
xy-106SER-R	cgaggggcagatcAGCaatcctgcgttcc
XY-106SER-F	ggaacgcaggattGCTgatctgcccctcg
xy-106TRP-R	cgaggggcagatcTGGaatcctgcgttcc
XY-106TRP-F	ggaacgcaggattCCAgatctgcccctcg
xy-106ASP-R	cgaggggcagatcGACaatcctgcgttcc
XY-106ASP-F	ggaacgcaggattGTCgatctgcccctcg
xy-106PHE-R	cgaggggcagatcTTCaatcctgcgttcc

XY-106PHE-F	ggaacgcaggattGAAgatctgcccctcg
xy-106TYR-R	cgaggggcagatcTATaatcctgcgttcc
XY-106TYR-F	ggaacgcaggattATAgatctgcccctcg
XY-106-R	ctcgaggggcagatcNNKaatcctgcgttccag
XY-106-F	ctggaacgcaggattMNNgatctgcccctcgag
xy-197LEU-R	gccttcgccaccgtgCTGgagatatccatcaac
XY-197LEU-F	gttgatggatatctcCAGcacggtggcgaaggc
xy-197MET-R	gccttcgccaccgtgATGgagatatccatcaac
XY-197MET-F	gttgatggatatctcCATcacggtggcgaaggc
xy-197ILE-R	gccttcgccaccgtgATCgagatatccatcaac
XY-197ILE-F	gttgatggatatctcGATcacggtggcgaaggc
xy-197SER-R	gccttcgccaccgtgAGCgagatatccatcaac
XY-197SER-F	gttgatggatatctcGCTcacggtggcgaaggc
xy-197THR-R	gccttcgccaccgtgACCgagatatccatcaac
XY-197THR-F	gttgatggatatctcGGTcacggtggcgaaggc
xy-197CYS-R	gccttcgccaccgtgTGCgagatatccatcaac
XY-197CYS-F	gttgatggatatctcGCAcacggtggcgaaggc
xy-197LYS-R	gccttcgccaccgtgAAGgagatatccatcaac
XY-197LYS-F	gttgatggatatctcCTTcacggtggcgaaggc
xy-197PHE-R	gccttcgccaccgtgTTCgagatatccatcaac
XY-197PHE-F	gttgatggatatctcGAAcacggtggcgaaggc
xy-197TYR-R	gccttcgccaccgtgTATgagatatccatcaac
XY-197TYR-F	gttgatggatatctcATAcacggtggcgaaggc
xy-197-R	gccttcgccaccgtgNNKgagatatccatcaac
XY-197-F	gttgatggatatctcMNNcacggtggcgaaggc
xy-200GLY-R	accgtggccgagataGGTatcaacgcctatctg
XY-200GLY-F	cagataggcgttgatACCtatctcggccacggt
xy-200ASN-R	accgtggccgagataAACatcaacgcctatctg
XY-200ASN-F	cagataggcgttgatGTTtatctcggccacggt
xy-200ILE-R	accgtggccgagataATCatcaacgcctatctg
XY-200ILE-F	cagataggcgttgatGATtatctcggccacggt
xy-200GLN-R	accgtggccgagataCAGatcaacgcctatctg
XY-200GLN-F	cagataggcgttgatCTGtatctcggccacggt
xy-200ARG-R	accgtggccgagataCGGatcaacgcctatctg
XY-200ARG-F	cagataggcgttgatGCCtatctcggccacggt
xy-200CYS-R	accgtggccgagataTGCatcaacgcctatctg
XY-200CYS-F	cagataggcgttgatGCAtatctcggccacggt
xy-200LYS-R	accgtggccgagataAAGatcaacgcctatctg
XY-200LYS-F	cagataggcgttgatCTTtatctcggccacggt
xy-200HIS-R	accgtggccgagataCACatcaacgcctatctg
XY-200HIS-F	cagataggcgttgatGTGtatctcggccacggt
xy-200GLU-R	accgtggccgagataGAGatcaacgcctatctg
XY-200GLU-F	cagataggcgttgatCTCtatctcggccacggt
xy-200TRP-R	accgtggccgagataTGGatcaacgcctatctg
XY-200TRP-F	cagataggcgttgatCCAtatctcggccacggt
XY-200-R	accgtggccgagataNNKatcaacgcctatctg
XY-200-F	cagataggcgttgatMNNtatctcggccacggt
XY-202ARG-F	ggttcagataggcCCGgatggatatctcg
XY-202ARG-R	cgagatatccatcCGGgcctatctgaacc
XY-202ASP-F	ggttcagataggcGTCgatggatatctcg
XY-202ASP-R	cgagatatccatcGACgcctatctgaacc

XY-202GLU-F	ggttcagataggcCTCgatggatatctcg
XY-202GLU-R	cgagatatccatcGAGgcctatctgaacc
XY-202HIS-F	ggttcagataggcGTGgatggatatctcg
XY-202HIS-R	cgagatatccatcCACgcctatctgaacc
XY-202TRP-F	ggttcagataggcCCAgatggatatctcg
XY-202TRP-R	cgagatatccatcTGGgcctatctgaacc
XY-202CYS-F	caggttcagataggcGCAgatggatatctcggc
XY-202CYS-R	gccgagatatccatcTGCgcctatctgaacctg
XY-202PHE-F	caggttcagataggcGAAgatggatatctcggc
XY-202PHE-R	gccgagatatccatcTTCgcctatctgaacctg
XY-202SER-F	caggttcagataggcGCTgatggatatctcggc
XY-202SER-R	gccgagatatccatcAGCgcctatctgaacctg
XY-202THR-F	caggttcagataggcCGTgatggatatctcggc
XY-202THR-R	gccgagatatccatcACGgcctatctgaacctg
XY-202TYR-F	caggttcagataggcGTAgatggatatctcggc
XY-202TYR-R	gccgagatatccatcTACgcctatctgaacctg
XY-202-F	ggttcagataggcMNNgatggatatctcg
XY-202-R	cgagatatccatcNNKgcctatctgaacc
xy-266ILE-R	ggtctcgaggcgATCgtcggcaacgac
XY-266ILE-F	gtcgttgccgacGATcgcctcgagacc
xy-266VAL-R	ggtctcgaggcgGTCgtcggcaacgac
XY-266VAL-F	gtcgttgccgacGACcgcctcgagacc
xy-266TYR-R	ggtctcgaggcgTATgtcggcaacgac
XY-266TYR-F	gtcgttgccgacATAcgcctcgagacc
xy-266GLN-R	ggtctcgaggcgCAGgtcggcaacgac
XY-266GLN-F	gtcgttgccgacCTGcgcctcgagacc
xy-266ALA-R	ggtctcgaggcgGCCgtcggcaacgac
XY-266ALA-F	gtcgttgccgacGGCcgcctcgagacc
xy-266GLU-R	ggtctcgaggcgGAGgtcggcaacgac
XY-266GLU-F	gtcgttgccgacCTCcgcctcgagacc
xy-266SER-R	ggtctcgaggcgTCCgtcggcaacgac
XY-266SER-F	gtcgttgccgacGGAcgcctcgagacc
xy-266LYS-R	ggtctcgaggcgAAGgtcggcaacgac
XY-266LYS-F	gtcgttgccgacCTTcgcctcgagacc
xy-266ASP-R	ggtctcgaggcgGATgtcggcaacgac
XY-266ASP-F	gtcgttgccgacATCcgcctcgagacc
XY-266-R	cggtctcgaggcgNNKgtcggcaacgact
XY-266-F	agtcgttgccgacMNNcgcctcgagaccg

Note: the mutation site is marked with capital letters.

Table S5. The primers of Q111E, Q104R, I199T.

Name of primers	Sequence (5' to 3')
xy-111E-R	atcctgcgttcGAGctcggcctgga
XY-111E-F	tccaggccgagCTCgaacgcaggat
XY-Q104R-R	gctgctcgaggggCGCatcgtcaatcctg
XY-Q104R-F	caggattgacgatGCGcccctcgagcagc
XY-I199T-R	caccgtggccgagACGtccatcaacgcct
XY-I199T-F	aggcgttgatggaCGTctcggccacggtg
xy-101104-R	accacggtgctgATCgaggggCGGatc
XY-101104-F	gatCCGcccctcGATcagcaccgtggt

Note: the mutation site is marked with capital letters.
